# An amelogenin-based peptide hydrogel promoted the odontogenic differentiation of human dental pulp cells

**DOI:** 10.1093/rb/rbac039

**Published:** 2022-06-17

**Authors:** Xinxin Li, Zhaoxia Yu, Shihui Jiang, Xiaohua Dai, Guanhua Wang, Yue Wang, Zhimou Yang, Jie Gao, Huiru Zou

**Affiliations:** State Key Laboratory of Medicinal Chemical Biology, Key Laboratory of Bioactive Materials, Ministry of Education, College of Life Sciences, Nankai University, Tianjin 300071, China; Tianjin Key Laboratory of Oral and Maxillofacial Function Reconstruction, Tianjin Stomatological Hospital, The Affiliated Stomatological Hospital of Nankai University, Tianjin 300041, China; School of Medicine, Nankai University, Tianjin 300071, China; Tianjin Key Laboratory of Oral and Maxillofacial Function Reconstruction, Tianjin Stomatological Hospital, The Affiliated Stomatological Hospital of Nankai University, Tianjin 300041, China; School of Medicine, Nankai University, Tianjin 300071, China; Tianjin Key Laboratory of Oral and Maxillofacial Function Reconstruction, Tianjin Stomatological Hospital, The Affiliated Stomatological Hospital of Nankai University, Tianjin 300041, China; School of Medicine, Nankai University, Tianjin 300071, China; Tianjin Key Laboratory of Oral and Maxillofacial Function Reconstruction, Tianjin Stomatological Hospital, The Affiliated Stomatological Hospital of Nankai University, Tianjin 300041, China; School of Medicine, Nankai University, Tianjin 300071, China; Tianjin Key Laboratory of Oral and Maxillofacial Function Reconstruction, Tianjin Stomatological Hospital, The Affiliated Stomatological Hospital of Nankai University, Tianjin 300041, China; School of Medicine, Nankai University, Tianjin 300071, China; State Key Laboratory of Medicinal Chemical Biology, Key Laboratory of Bioactive Materials, Ministry of Education, College of Life Sciences, Nankai University, Tianjin 300071, China; State Key Laboratory of Medicinal Chemical Biology, Key Laboratory of Bioactive Materials, Ministry of Education, College of Life Sciences, Nankai University, Tianjin 300071, China; Tianjin Key Laboratory of Oral and Maxillofacial Function Reconstruction, Tianjin Stomatological Hospital, The Affiliated Stomatological Hospital of Nankai University, Tianjin 300041, China; School of Medicine, Nankai University, Tianjin 300071, China

**Keywords:** amelogenin, peptide hydrogel, odontogenic differentiation, human dental pulp cells

## Abstract

Amelogenin can induce odontogenic differentiation of human dental pulp cells (HDPCs), which has great potential and advantages in dentine-pulp complex regeneration. However, the unstability of amelogenin limits its further application. This study constructed amelogenin self-assembling peptide hydrogels (L-gel or D-gel) by heating-cooling technique, investigated the effects of these hydrogels on the odontogenic differentiation of HDPCs and explored the underneath mechanism. The critical aggregation concentration, conformation, morphology, mechanical property and biological stability of the hydrogels were characterized, respectively. The effects of the hydrogels on the odontogenic differentiation of HDPCs were evaluated via alkaline phosphatase activity measurement, quantitative reverse transcription polymerase chain reaction, western blot, Alizarin red staining and scanning electron microscope. The mechanism was explored via signaling pathway experiments. Results showed that both the L-gel and D-gel stimulated the odontogenic differentiation of HDPCs on both Day 7 and Day 14, while the D-gel showed the highest enhancement effects. Meanwhile, the D-gel promoted calcium accumulation and mineralized matrix deposition on Day 21. The D-gel activated MAPK-ERK1/2 pathways in HDPCs and induced the odontogenic differentiation via ERK1/2 and transforming growth factor/smad pathways. Overall, our study demonstrated that the amelogenin peptide hydrogel stimulated the odontogenic differentiation and enhanced mineralization, which held big potential in the dentine-pulp complex regeneration.

## Graphical Abstract

**Figure:**
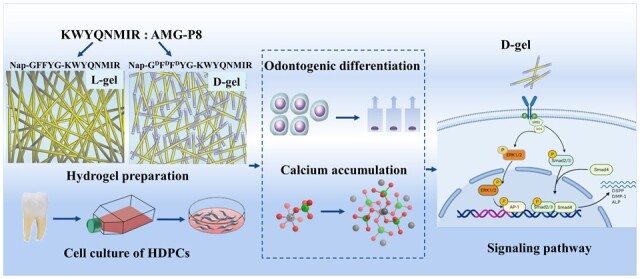

## Introduction

Dentine-pulp complex is a highly specialized tissue for protecting the vital teeth. It can be damaged due to dental caries, trauma or other problems [1, 2]. The regeneration of the dentine-pulp complex is of paramount importance to regain tooth vitality. However, there is no ideal system that can not only enable the proliferation and differentiation of relevant progenitor cells but also mimick the natural extracellular environment and modulate relative responses [3–5].

Amelogenins are enamel matrix proteins (EMPs) currently used to treat bone defects in periodontal surgery [6]. These proteins represent about 90% of the EMPs and contain many isoforms which are responsible for the biological effects of EMPs. Recent studies have highlighted several amelogenin-derived peptides, such as leucine-rich amelogenin peptide (LRAP), tyrosine-rich amelogenin peptide and C-terminal amelogenin telopeptide, in bone tissue engineering, mineralization and dentinal tubule occlusion [7–10]. Interestingly, these peptides seem to maintain or even enhance the biological activity of the full-length amelogenin protein. However, the therapeutic efficacy of the bioactive peptides is often reduced because of the degradation or conformation changes. It is very necessary to develop new formulations or delivery systems of therapeutic peptides that can boost their full effects.

Several hydrogels have been investigated for potential regenerative endodontic applications due to their injectable, high-water content, controllable porosity and mechanical properties [11–13]. The supramolecular hydrogel formed by self-assembled bioactive peptides can significantly increase the stability and bioavailability [14, 15]. For example, our previous research found that an insulin-like growth factor-I (IGF-1) self-assembled peptide supramolecular nanofibers enhanced the bioactivity of the IGF-1 peptides [16]. Xia *et al.* [17] also showed that RGD- and vascular endothelial growth factor-mimetic peptide scaffolds supported dentine bridge formation. In recent years, 2-Naphthaleneacetic acid (Nap) has been used as an aromatic end-capping group and Nap-GFFY (G^D^F^D^F^D^Y) has been used as self-assembly motifs in multiple studies due to the existence of multiple benzene rings, with excellent self-assembly properties [18–20]. These strategies all have great potentials for dental pulp tissue engineering and regeneration, but still need further study.

Inspired by these pioneering works, an amelogenin-based peptide (amino sequence KWYQNMIR, namely AMG-P8) was conjugated with Nap-GFFY or Nap-G^D^F^D^F^D^Y to form hydrogels (L-gel or D-gel) in this study, which may greatly improve the biological stability of amelogenin peptide. The biological effects of these peptide hydrogels on the odontogenic differentiation of human dental pulp cells (HDPCs) were evaluated. Results showed that both the L-gel and D-gel enhanced the odontogenic differentiation of HDPCs, while the D-gel showed the highest enhancement effects. The D-gel was confirmed to activate mitogen-activated protein kinase-extracellular regulated protein kinases (MAPK-ERK1/2) pathways in HDPCs, and induce the odontogenic differentiation via ERK1/2 and transforming growth factor (TGF)/smad pathways. Therefore, our study offered a very effective peptide hydrogel strategy, which held big potential in the dentine-pulp complex regeneration.

## Materials and methods

### Synthesis of amelogenin and self-assembled peptides

The amelogenin peptides (KWYQNMIR) and self-assembled peptides (Nap-GFFY-G-KWYQNMIR and Nap-G^D^F^D^F^D^Y-G-KWYQNMIR) were synthesized by standard solid-phase peptide synthesis method. Firstly, the C-terminal of the first amino acid was conjugated on the resin. Twenty percent piperidine in anhydrous N, N′-dimethylformamide was used during deprotection of Fmoc group. O-Benzotriazol-1-yl-N, N, N′,N′- tetramethyluronium hexafluorophosphate was used as coupling reagent. Then, 95% trifluoroacetic acid containing 2.5% H_2_O and 2.5% trimethylsilane was used to cleave peptides derivative from resin. Diethylether was poured into filtrate performed by rotary evaporation. The solid was dried by vacuum pump to gain desirable compounds. The crude products were separated by high-performance liquid chromatography with methanol and H_2_O containing 0.05% trifluoroacetic acid as eluents. Finally, the pure peptide powder was obtained by freeze-drying method.

### Amelogenin peptide hydrogel preparation

Nap-GFFY-G-KWYQNMIR or Nap-G^D^F^D^F^D^Y-G-KWYQNMIR (1.88 mg each) was added to a glass vial containing 500 μl of H_2_O, respectively. The suspension was heated with gentle shaking to prevent bumping. A transparent solution was obtained when the temperature reached about 90°C. Na_2_CO_3_ solution (1 M) was used to adjust the final pH value to 7.4 (about 2 equivalent to the peptide). After the pH of the solution was adjusted, the Nap-GFFY-G-KWYQNMIR or Nap-G^D^F^D^F^D^Y-G-KWYQNMIR was ready to form hydrogel (L-gel or D-gel, respectively) after being kept at room temperature (22–25°C) for within 5 min.

### Characterization of the amelogenin peptide hydrogel

The ultrastructure of the amelogenin peptide hydrogel was observed using transmission electron microscope (TEM). A drop of the hydrogel was placed on a carbon-coated copper grid and dried at room temperature. The sample was stained with a saturated uranyl acetate solution, placed in a desiccator overnight and then analyzed.

The critical aggregation concentration (CAC) values of the AMG-P8, L-gel and D-gel were determined by dynamic light scattering (DLS). Solutions containing different concentrations of compound were tested and the light scattering intensity was recorded for each concentration analyzed.

The mechanical properties of the L-gel and D-gel were detected by the rheometer. Hydrogels at the concentration of 3 mM were subjected to a rheological test (AR 2000ex system, TA Instruments, New Castle, DE, USA). The parallel plates used for the test were 25 mm and the gap during the experiment was 500 μm.

The circular dichroism spectrum of the AMG-P8, L-gel and D-gel at the concentration of 2 mM was acquired on a circular dichroism spectroscopy (Bio-Logic MOS-450, France). The wavelength ranged from 180 to 260 nm. The acquisition period was 1 s and the step was 1 nm. The resultant circular dichroism spectra were acquired by subtracting the solvent background.

The Zeta potential of the hydrogel was determined by the DLS method using the standard protocol. All the solutions were filtered by 0.22 μm filter membrane. The zeta potential was detected with the ZETAPALS/BI-200SM system. All experiments were repeated for three times.

### Cell culture

The healthy premolars and third molars extracted for orthodontic or other reasons were collected from patients (14–22 years old) visited Tianjin Stomatological Hospital. This study had been approved by the Ethics Committee of Tianjin Stomatological Hospital (Approval number: 2018012). The informed consent was obtained from each patient. The dental pulp tissue was dissected within 4 h after tooth extraction, minced into 1 mm^3^ pieces with sterile scissors and transferred to a cell culture flask containing alpha-modified Eagle’s medium (α-MEM, Gibco, California, USA) supplemented with 10% fetal bovine serum (FBS, Gibco, New York, NY, USA), 100 U/ml penicillin and 100 μg/ml streptomycin (PS, Sigma, California, USA). The cells were cultured in a 5% CO_2_ incubator at 37°C. The HDPCs at 3–4 passage were used in this study.

### Quantitative reverse transcription polymerase chain reaction

The HDPCs were seeded in the 6-well plates at a density of 1 × 10^5^ cells/well. The 10% FBS medium in the presence or absence of the AMG-P8, L-gel, D-gel (1 µM) was added to stimulate cells for 7 and 14 days. Cells treated with hydrogels without amelogenin peptide were served as another two blank control groups. Meanwhile, cells treated with equivalent amount of whole EMPs were served as positive control groups. The culture medium was refreshed every 3 days. For the quantitative reverse transcription polymerase chain reaction (qRT-PCR) analysis, the total RNA was extracted by the MiniBEST Universal RNA Extraction Kit (Takara, Tokyo, Japan). The RNA concentration and purity were measured by an ultraviolet-visible spectrophotometer (NanoDrop Wilmington, USA). The RNA was subjected to reverse transcription using the PrimeScript™ RT reagent Kit (Takara, Tokyo, Japan). The qRT-PCR was performed with the TB Green™ Premix Ex Taq™ II (Takara, Tokyo, Japan) using a LightCycler^®^ 480 II (Roche, Basel, Switzerland). The relative mRNA expression level of dentine sialophosphoprotein (DSPP), dentine matrix protein 1 (DMP-1), collagen type 1 (COL-1), runt-related transcription factor 2 (RUNX-2) were calculated by the 2^−ΔΔCt^method.

### Western blot analysis

The HDPCs (2 × 10^5^) were treated under different conditions for different time points. The EMPs (1 µM) were also added to the HDPCs as an extra control group. Then the cells were harvested and lysed in 200 µL lysis buffer (Beyotime, Shanghai, China) and centrifuged (12 000 rpm for 10 min, at 4°C). The protein concentration was evaluated with a BCA kit (Solarbio, Beijing, China). The protein was mixed with 5× loading buffer and heated at 95°C for 5 min, separated by precast PAGE gel (Beyotime, Shanghai, China) and transferred to polyvinylidene fluoride membranes. After blocked at room temperature for 15 min, then incubated with the primary antibody (DSPP, DMP-1, Smad2/3, c jun, p-c jun, c fos, p-c fos, c jun B) (Santa Cruz, California, USA), [p-ERK1/2, ERK1/2, alkaline phosphatase (ALP), p-Smad2/3, GAPDH] (Beyotime, Shanghai, China). Then, the membranes were incubated with the goat anti-rabbit IgG-HRP secondary antibody or goat anti-mouse IgG-HRP secondary antibody for 1 h, respectively. Then, an ultrasensitive chemiluminescence kit (Beyotime, Shanghai, China) was used to detect the protein bands. A chemiluminescence imager (Bio-Rad, California, USA) was used to scan the protein bands. The Image Lab software was used to analyze the relative protein expression levels.

### ALP activity

The HDPCs (1 × 10^5^ cells/well) in the six well plates were cultured for 14 days. The cells were lysed using the cell lysis buffer. The cell debris were removed by centrifugation. The ALP activity of the lysate was detected by an ALP activity kit (Jiancheng, Nanjing, China) at 520 nm wavelength. The ALP activity quantification was normalized by protein concentration according to the manufacturer’s instructions.

### Alizarin red staining

The HDPCs (1 × 10^5^ cells/well) in the six well plates were treated with basal medium, AMG-P8, L-gel, D-gel, EMPs for 21 days. After fixed in the 4% paraformaldehyde for 15 min, the mineralized nodules were stained with 1% Alizarin Red (Solarbio, Beijing, China) for 10 min and washed in double-distilled water to eliminate floating colors. Images were captured with a Ti-S microscope (Nikon, Japan). For quantitative analysis of calcified nodules, 10% cetylpyridinium chloride (CPC; Sigma, USA) was used to dissolve Alizarin red. One milliliter of 10% CPC per well was added and shook for 30 min. The absorbance value was measured at 560 nm using a multifunctional microplate reader (Tecan Ltd., Männedorf, Switzerland).

### Scanning electron microscopy and energy-dispersive spectrometry

Cell culture was the same as the Alizarin red staining experiment. The cells were fixed with 4% paraformaldehyde at 4°C for 24 h, and then dehydrated in a series of increasing concentrations of ethanol (30%, 50%, 70%, 90%, 95% and 100% v/v), dried and coated with gold. Then the cells were observed under a scanning electron microscope (FEI QUANTA 200, FEI, Holland). The mineralized nodules were randomly selected and their weight percent and the atomic percent content of the main elements (C, O, Ca and P) were determined by the energy-dispersive X-ray spectroscopy (EDX) qualitative analysis.

### Confocal fluorescence laser scanning microscopy

The HDPCs (5 × 10^4^ cells) were seeded in the 35 mm laser confocal dishes for 24 h and starved in the serum-free medium for 24 h. Then they were randomly divided into four groups, which were the control group, the D-gel group, the D-gel+U0126 group and the U0126 group. The cells were first treated with 10 µM p-ERK1/2 inhibitor U0126 in the D-gel+U0126 group and the U0126 group for 1 h. Next, 1 µM D-gel diluted by serum-free medium was added to stimulate cells in the D-gel group and the D-gel+U0126 group for 1 h. The cells were fixed in 4% cold formaldehyde for 15 min. Then the cells were covered with 100% cold methanol and incubated at –20°C for 10 min. After washed with PBS for 5 min and blocked with blocking buffer (Beyotime, Shanghai, China) at room temperature for 10 min, the cells were incubated with a 1:200 dilution of anti-rabbit ERK1/2 (Beyotime, Shanghai, China) and anti-rabbit p-ERK1/2(Cell Signaling Technology, USA) at 4°C overnight. A 1:200 dilution of 488-conjugated goat anti-rabbit IgG (H + L) or Cy3 conjugated goat anti-rabbit IgG (H + L) (Proteintech Group, USA) was used as the secondary antibody for 1 h. The cell nucleus was stained with DAPI for 5 min (Beyotime, Shanghai, China). The images were captured using a confocal microscope (Leica, Germany).

### Statistical analysis

Statistical analyses were performed using SPSS software (Version 22, SPSS Inc, USA). All datasets had been tested for normal distribution using the Kolmogoroff–Smirnov test or Shapiro–Wilk test and for homogeneity of variances using Levene test. All assays were performed in triplicate for each condition, and each experiment was repeated at least three times. The means and standard deviations (SD) were recorded and analyzed by ANOVA and the Student’s *t*-test. *P *<* *0.05 was considered as statistically significant.

## Results

### AMG-P8 synthesis, hydrogel preparation and characterization

**Figure 1.**
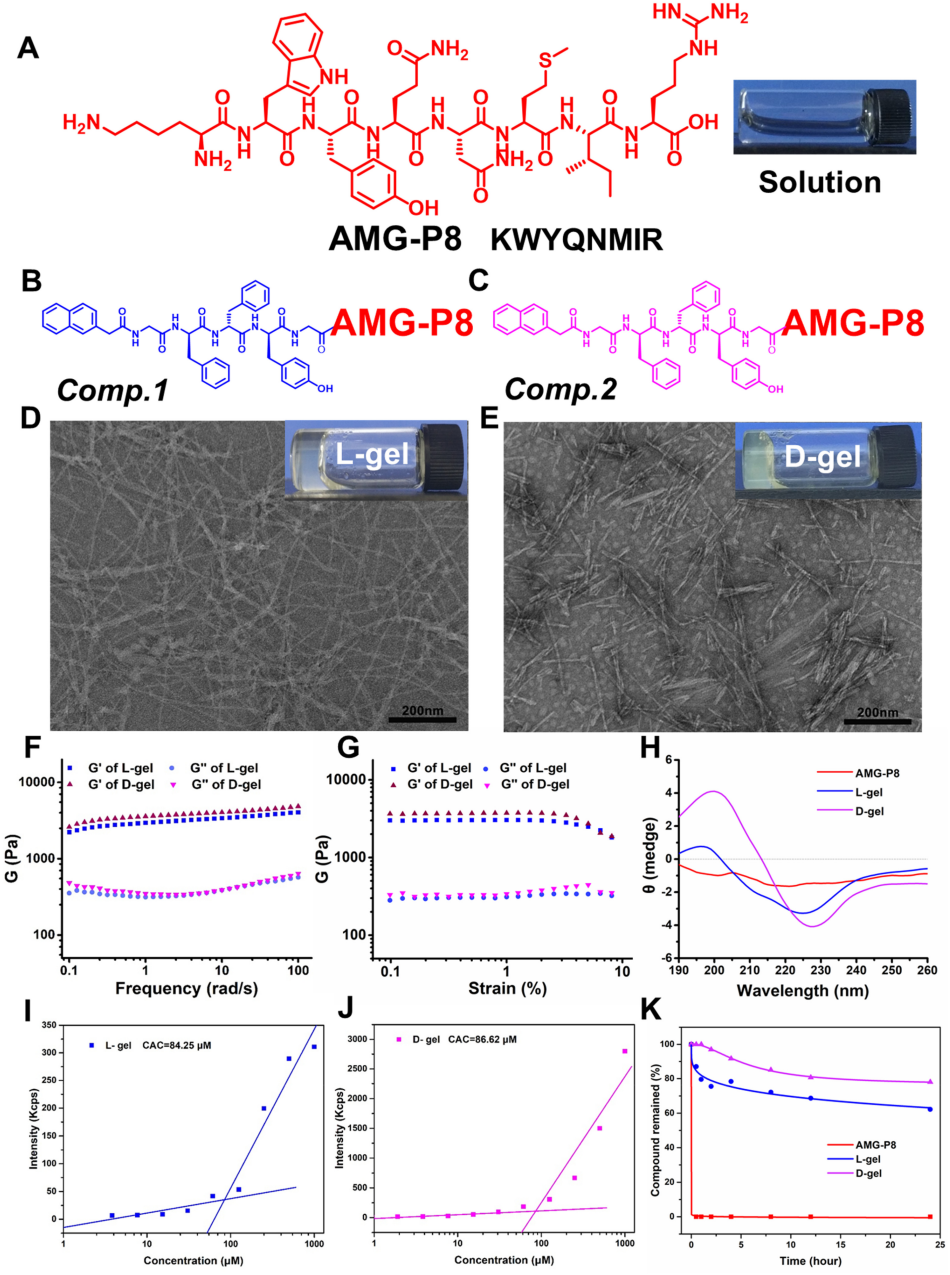
Chemical structures of (**A**) AMG-P8 (KWYQNMIR); (**B**) Comp.1 (Nap-GFFYGKWYQNMIR) and (**C**) Comp.2 (Nap-G^D^F^D^F^D^YGKWYQNMIR). TEM and optical images of (**D**) L-gel and (**E**) D-gel. Dynamic frequency and strain sweep measurements of L-gel and D-gel (**F**, **G**). (**H**) Circular dichroism spectra of L-gel and D-gel. Determination of CAC values of L-gel (**I**) and D-gel (**J**). (**K**) Compound remaining of the AMG-P8, L-gel and D-gel for an initial concentration of 1.0 mM incubated with 0.1 mg/ml of proteinase K for 24 h.

The AMG-P8 peptide had good solubility. Both Nap-GFFY-G-KWYQNMIR (*Comp.* 1) and Nap-G^D^F^D^F^D^Y-G-KWYQNMIR (*Comp.* 2) could form hydrogels by heating and cooling, namely L-gel and D-gel (Fig. 1A–C). The ultrastructures of the L-gel and D-gel were both nanofibers (Fig. 1D and E). The mechanical properties of the L-gel and D-gel remained relatively constant (Fig. 1F and G). The D-gel had a secondary structure similar to β-sheet conformation (Fig. 1H). The CAC values of the AMG-P8, L-gel and D-gel were 354.91, 84.25 and 86.62 μM, respectively (Fig. 1I and J). The proteinase K experiments showed that the AMG-P8 was rapidly hydrolyzed within 30 min. That was the result of an initial concentration of 1.0 mM AMG-P8 incubated with 0.1 mg/ml of proteinase K for 24 h, whereas the L-gel and D-gel still retained 62.21% and 78.06% of the compound, respectively, after 24 h (Fig. 1K).

### Effects of AMG-P8 hydrogel on the odontogenic differentiation of HDPCs

**Figure 2.**
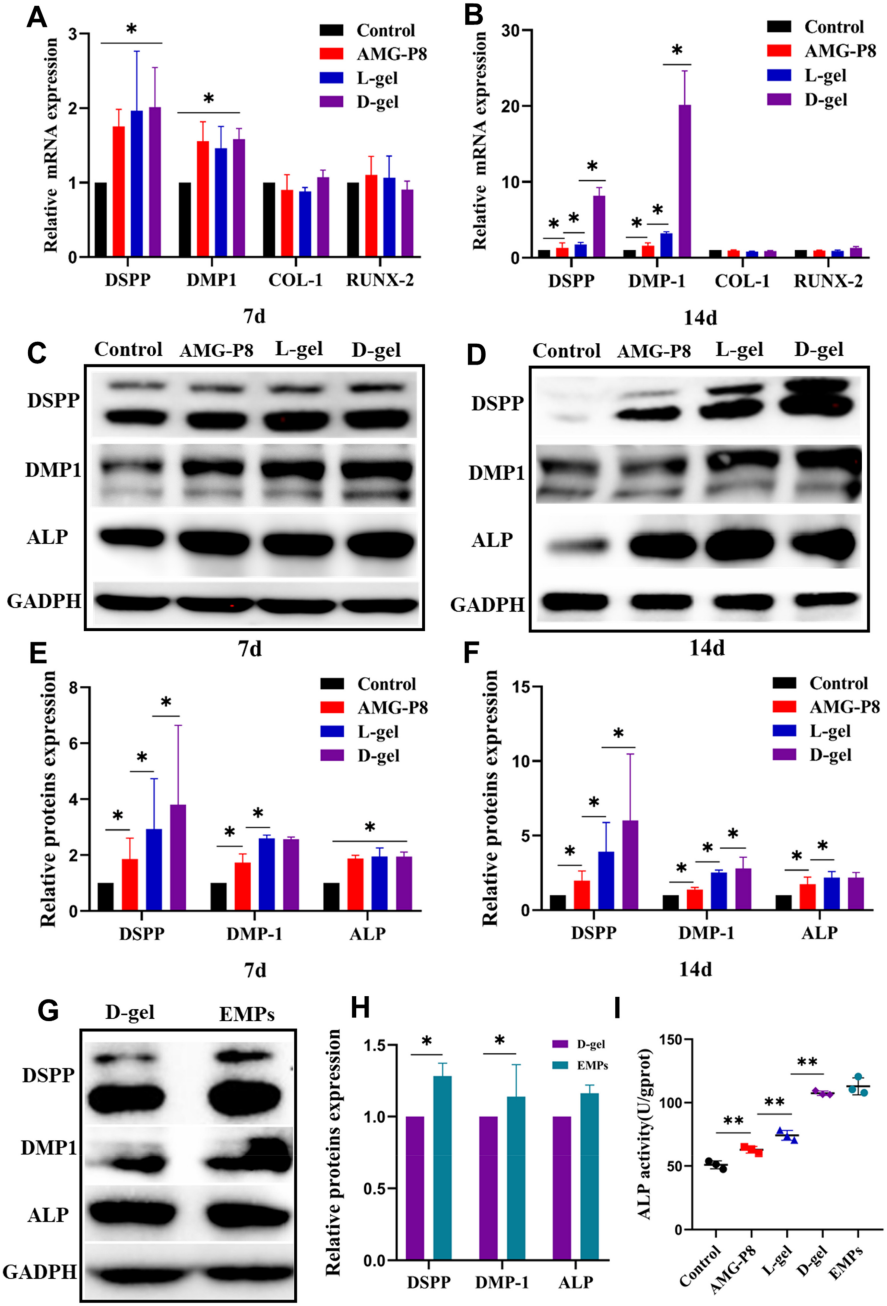
Effects of AMG-P8 and supramolecular hydrogels on the odontogenic differentiation of HDPCs. (**A, B**) The relative mRNA expression of DSPP, DMP-1, RUNX-2, COL-1 on Day 7 and Day 14 (*n* = 3). (**C, D**) The protein expression of DSPP, DMP-1 and ALP on Day 7 and Day 14 (*n* = 3). (**E, F**) The quantitative protein expression analysis. (**G, H**) The protein expression and quantitative analysis of DSPP, DMP-1 and ALP in the D-gel and EMPs groups on Day 14. (I) ALP activity of all groups. The data were presented as means ± SD. *P <* *0.05, **P < 0.01.

*DSPP* and *DMP-1* were upregulated in the AMG-P8, L-gel and D-gel groups at Day 7 (*P *<* *0.05). The difference was more obvious at Day 14 (*P *<* *0.05). Surprisingly, *DSPP* and *DMP-1* mRNA expression levels in the D-gel group were 8- and 20-fold than that of the control group, respectively (*P *<* *0.05) (Fig. 2A and B). Nap-GFFY or Nap-G^D^F^D^F^D^Y hydrogel without amelogenin peptide had no obvious effects on HDPCs.

The protein expression of DSPP, DMP-1 and ALP was remarkably increased in the AMG-P8, L-gel and D-gel groups (*P *<* *0.05). The D-gel even achieved nearly the same effects as the whole EMPs (Fig. 2G and H).

The ALP activity on Day 14 was slightly enhanced by the AMG-P8 and significantly increased by the L-gel and D-gel (Fig. 2I). Among them, the D-gel group got the most extraordinary results (*P *<* *0.05).

### AMG-P8 hydrogel promoted the mineralization of HDPCs

The HDPCs cultured in basal medium for 21 days generated a few small calcified nodules, while the AMG-P8 treated group accumulated more small calcified nodules. Interestingly, orange-red mineralized nodules in the D-gel group were extremely numerous and huge which were similar to that of the EMPs group. The quantitative result showed that the OD value of the D-gel treated group was 3-fold higher than the control group (*P *<* *0.001) and was about 80% of the EMPs group (*P < *0.05).

**Figure 3.**
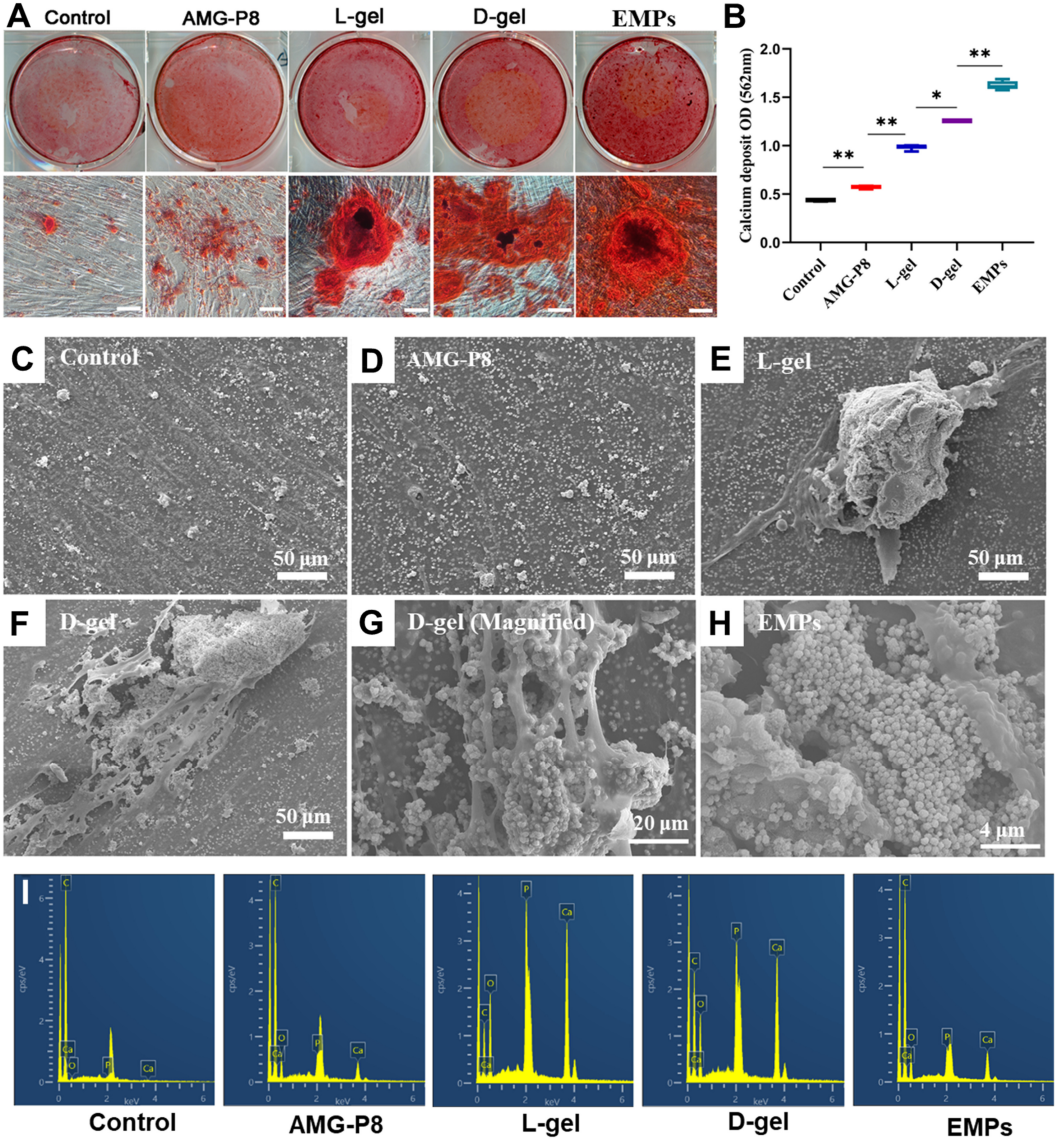
(**A**) Effects of AMG-P8 and supramolecular hydrogels on the mineralization of HDPCs. Alizarin red staining on Day 21 (*n* = 3). Scale bar = 100 μm. (**B**) The quantitative analysis of alizarin red staining. (**C–F**) The microscopic morphology of mineralized nodules on Day 21. Scale bar = 50 μm. (**G**) HDPCs in D-gel group. Scale bar = 20 μm. (**H**) HDPCs in EMPs group. Scale bar = 4 μm. (**I**) Calcium–phosphorus (Ca/P) ratio of all groups (*n* = 3). The data were presented as means ± SD. *P < 0.05, **P <* *0.01.

Scanning electron microscopy (SEM) showed that mineralized crystals were fused into calcium spheres, which scattered in the control and AMG-P8 group. While the diameter of calcified clusters was dozens or even hundreds of times larger in the hydrogel group than that of the control or AMG-P8 group. The HDPCs around the D-gel exhibited cytoplasmic elongation and produced more mineralized nodules than the L-gel and the EMPs. The Ca/P ratios of the L-gel, D-gel and EMPs groups reached 1.49 ± 0.13, 1.65 ± 0.09 and 1.46 ± 0.21, respectively (Fig. 3I).

### D-gel activated MAPK-ERK 1/2 pathways in HDPCs

**Figure 4.**
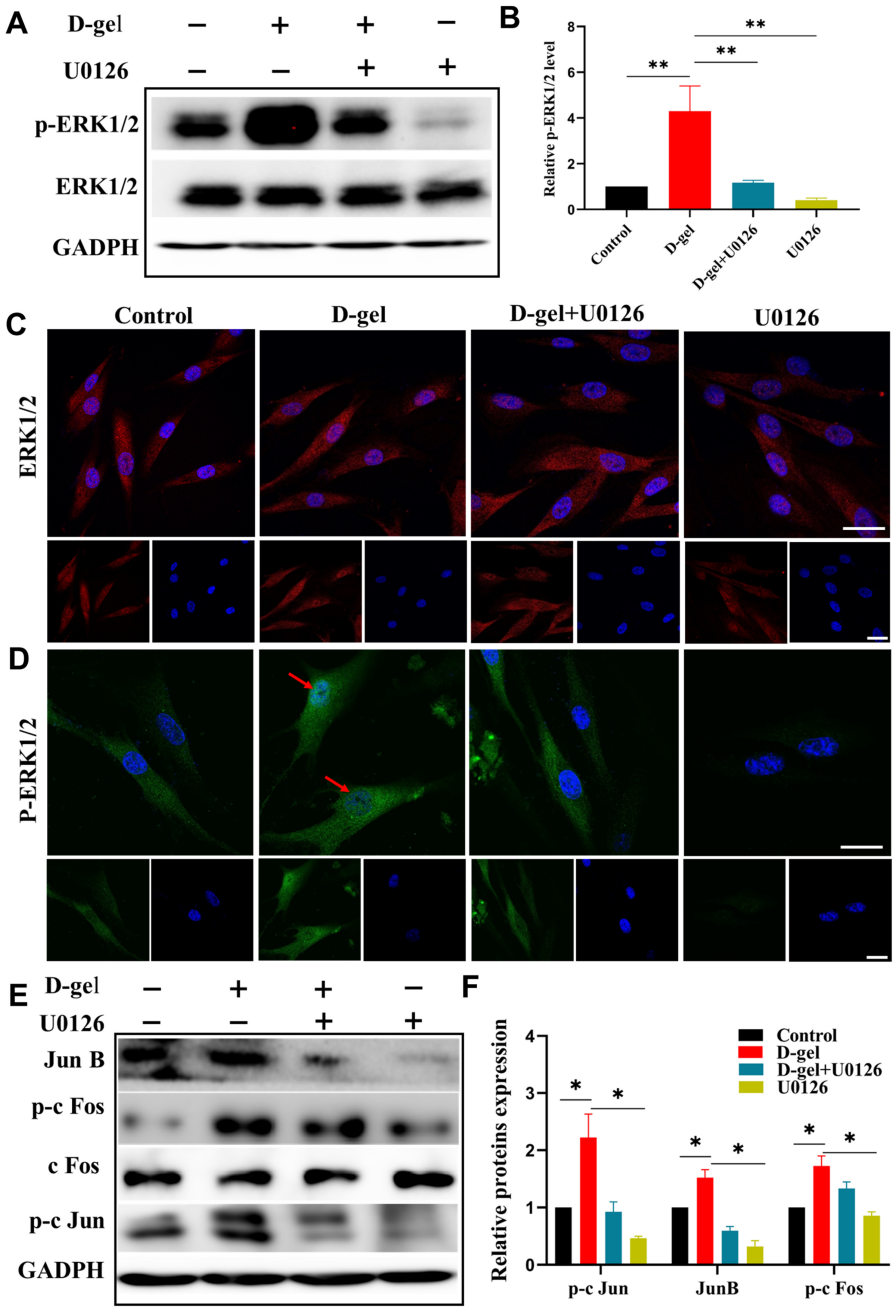
D-Gel activated ERK 1/2 pathways in HDPCs. (**A**) Western blot analysis of p-ERK1/2 and ERK1/2 from HDPCs pretreated with specific inhibitors U0126 for 1 h followed by stimulation with D-gel for 1 h (*n* = 3). (**B**) The quantitative protein analysis of p-ERK1/2. (**C, D**) Representative images of ERK1/2 and p-ERK1/2 immunofluorescence staining of HDPCs after being treated with D-gel for 1 h, nuclei were counter stained with DAPI. Scale bar = 20 μm (*n* = 3). (**E, F**) The protein expression and quantitative result of AP-1 family (Jun B, p-c Jun, c Fos and p-c Fos) after being treated with D-gel for 1 h (*n* = 3). The data were presented as means ± SD. *P <* *0.05, **P <* *0.01.

The protein level of phospho-ERK1/2 was remarkably upregulated after 1 h incubation in the D-gel group compared with the control group (*P *<* *0.05) (Fig. 4A and B). This effect was specifically blocked by U0126 (*P *<* *0.05). Immunofluorescence analysis of ERK1/2 (Fig. 4C) and phospho-ERK1/2 (Fig. 4D) further confirmed that the highly expressed p-ERK1/2 stimulated by D-gel moved to the nucleus. ERK was distributed in the cytoplasm and nucleus, and its expression level was consistent with the western blot result. Moreover, the downstream of ERK1/2 pathway, AP-1 family (Jun B, p-c Jun, c Fos and p-c Fos) were activated after being treated with D-gel for 1 h and suppressed by U0126 (Fig. 4E and F) (*P *<* *0.05).

### D-gel induced odontogenic differentiation of HDPCs via ERK 1/2 and TGF-β/smad signaling pathway

**Figure 5.**
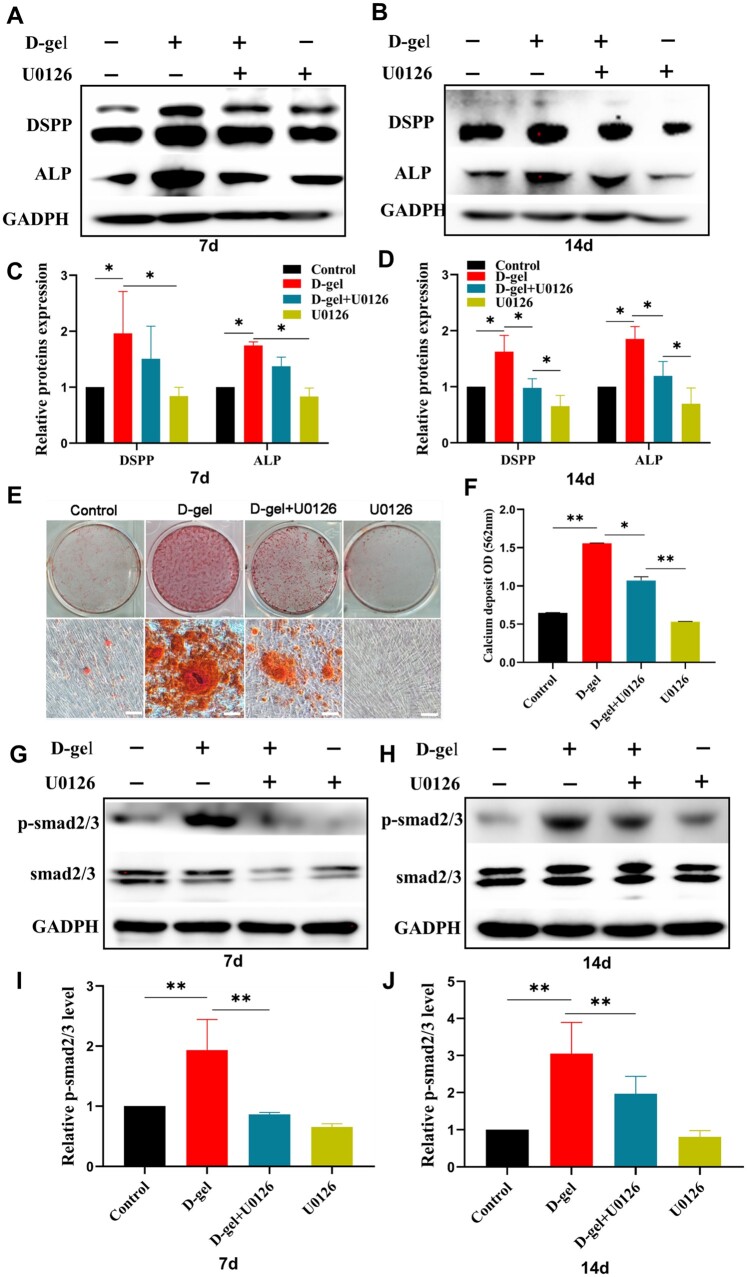
D-Gel enhanced HDPCs odontogenic differentiation through MAPK-ERK1/2 and TGF-β/smad signaling pathway. (**A, B**) The protein expression of DSPP and ALP of HDPCs treated with D-gel and U0126 for 7 and 14 days (*n* = 3). (**C, D**) The quantitative protein analysis of DSPP and ALP. (**E**) Alizarin red staining of HDPCs treated with D-gel and U0126 for 21 days (*n* = 3). (**F**) The quantitative analysis of alizarin red staining. (**G, H**) The protein expression of smad2/3 and p-smad2/3 after being treated with D-gel and U0126 for 7 and 14 days (*n* = 3). (**I, J**) The quantitative protein analysis of p-smad2/3. The data were presented as means ± SD. *P < 0.05, **P < 0.01.

U0126 suppressed the highly expression of DSPP and ALP induced by D-gel (*P *<* *0.05). In addition, mineralized nodules remarkably decreased in the D-gel+U0126 group (Fig. 5E and F). p-Smad2/3 was enhanced in the D-gel group when compared with the control group on Day 7 (1.93-fold increase) and Day 14 (3.05-fold increase), while the presence of U0126 decreased their expression level (*P *<* *0.05).

## Discussion

Amelogenin, the most abundant EMP, plays critical roles during enamel and dentine matrix deposition and mineralization. Full-length amelogenin influences the differentiation of HDPCs, which is potentially useful in pulp capping procedures [21]. It can also signal cells to enhance apex formation in nonvital immature teeth and promote soft connective tissue regeneration. Mounir *et al.* [22] compared recombinant DNA-produced amelogenin protein with calcium hydroxide in a study of immature apex closure conducted in 24 young mongrel dogs. Results confirmed that after 1 month, calcified tissue formed at the apical foramen and pulp chamber containing soft connective tissue and hard tissue in amelogenin-treated canals. After 3 and 6 months, apical tissue is functionally attached to bone by a periodontal ligament in amelogenin-treated canals. While calcified apical tissue was poorly formed in the calcium hydroxide group, and soft connective tissue within the pulp chamber was not observed. Amelogenin proteins are very useful. However, these proteins have different isoforms and splicing products, leading to complicated and unstable effects.

Previously we found an amelogenin peptide (namely AMG-P8) can stimulate odontogenic differentiation of HDPCs. However, the culture medium contained this peptide need to be renewed every 3 days. In another words, the effects could not last a long time. Therefore, we tried self-assembling peptide technique to form amelogenin peptide hydrogels with the expect of enhancing the stability and bioactivity of the peptide. Such technique might be very useful in dentine-pulp complex regeneration in future.

In this study, Nap-GFFY and Nap-G^D^F^D^F^D^Y were covalently linked to the peptide. The circular dichroism spectrum results showed that the D-gel exhibited β-sheet secondary structure while AMG-P8 peptide alone was randomly coiled. A number of studies had shown that hydrogels containing β-sheets exhibited amazing recombination ability and achieved outstanding stability both *in vivo* and *in vitro* [23, 24]. Cells generally lacked the enzymes to hydrolyze the D-configuration amino acids. Therefore, academics introduced it into peptides to improve the stability [25]. Our results also confirmed that D-gel was more resistant to enzymatic hydrolysis than L-gel. HDPCs cultured with AMG-P8 and hydrogels were also investigated at different time points to verify the stability of hydrogels *in vitro*. Result showed that the amelogenin hydrogels kept stable in culture dishes with cells clustering around after 14 days. After 18 days of culture, opaque nodules were observed in culture dishes, which were confirmed as mineralized nodules by alizarin red staining subsequently.

In this study, the relationship between AMG-P8 and EMPs was also considered. EMPs and recombinant LRAP have previously been confirmed to promote DSPP and DMP-1 expression of HDPCs and increase the ALP activity [26, 27]. This study showed that D-gel significantly upregulated the mRNA and protein expression level of DSPP and DMP-1 than L-gel and AMG-P8. The ALP activity in the D-gel group also increased significantly. Our results clearly indicated that D-gel enhanced the biological activity of AMG-P8 and promoted the odontogenic differentiation of HDPCs.

Previous studies had revealed that EMPs could promote mineralization of HDPCs [28]. As shown in Fig. 3, the D-gel significantly increased the number and size of mineralized nodules. Interestingly, SEM results showed that mineralized nodules presented in hydrogel group were obviously different. The mineralized nanospheres aggregated in the hydrogel and formed huge nodules which were proved to be similar to hydroxyapatite by EDX analysis. As it was well known that the first core stage of biomineralization was to provide nucleation sites, mineralization templates and growth space after odontoblasts secreting organic matrix and self-assemble in an orderly manner. The 3D network formed by D-gel might simulate the function of extracellular matrix, attract the deposition of calcium and phosphorus ions and provide growth space, thereby determining the scaffold microstructure and cell interactions. Therefore, HDPCs in the D-gel group exhibited cytoplasmic elongation and produced more mineralized nodules than the L-gel group. Chen *et al.* [28] revealed that there was a close relevance between cytoskeleton assembly structure and osteogenic differentiation induced by stiffness of mineralized hydrogel. The hydrogel with higher stiffness induced higher cell spreading and faster cytoskeleton assembly, consisting with higher osteogenic differentiation. Other studies also confirmed that self-assembling peptide hydrogels could simulate extracellular matrix secretion and provide structural support for cells [29]. In this study, compared with protein solutions that lacked mechanical properties, D-gel had greater advantages in inducing cell adhesion and matrix formation and mineralization.

EMPs and some amelogenin peptides regulated cellular behavior through the ERK1/2 signaling pathway [30, 31]. The p-ERK1/2 level was notably upregulated with D-gel treatment and reduced with U0126 block. Confocal fluorescence laser scanning microscopy results showed that the upregulated p-ERK1/2 transferred to nucleus. Simultaneously, the downstream of ERK1/2 pathway, AP-1 transcription factor family, including p-c fos, p-c jun, c jun B were also activated. AP-1 was one of the most important transcription factors to regulate cell growth and differentiation [32]. Both c Fos and c Jun were strongly expressed in odontoblasts of tooth germs. And there were AP-1 binding sites on genes such as DSPP and DMP-1 [33, 34]. Therefore, we speculated that D-gel might activate ERK1/2 and regulate the expression of c Fos, c Jun and Jun B.

Smad2 and Smad3 were specific signal transduction molecules, which could phosphorylate the type I receptor of TGF-β and initiate transcription of ALP, COL-1, DSPP, etc. [35]. There was widespread crosstalk between the MAPK-ERK1/2 pathway and the TGF-β/smad pathway. The kinase of MAPK-ERK1/2 could enhance the phosphorylation, nuclear translocation and transcriptional activities of Smad2/3. TGF-β itself could also activate ERK1/2 signal, and Smad2/3 could interact with AP-1 to regulate target gene transcription. A study had found that strong activity of Smad2/3 was observed in the odontoblast-like cells from the newly formed reparative dentine [36]. The regulation of teeth development by TGF-β might be coregulated by p-Smad2/3 and p-ERK1/2. We found that the p-Smad2/3 expressions significantly increased in the D-gel group and were repressed by U0126, which suggested that D-gel might promote the odontogenic differentiation and mineralization of HDPCs through the ERK/AP-1 signaling pathway. TGF-β/smad pathway might involve in and had a crosstalk with ERK1/2 pathway.

A limitation of this study was that the concentration of the hydrogel used here was relatively low, which was conducive to cell connection but difficult in establishing a suitable 3D environment for cell growth. How to further modify D-gel to present ideal mechanical properties, and adapt to the anatomical shape of pulp cavity after injection might be the future direction. In addition, our research showed that D-gel might activate multiple pathways. Further investigations and attempts were needed to interpret the comprehensive mechanisms and other signaling pathways underneath the effects of the supermolecular hydrogel.

## Conclusion

In summary, we developed an amelogenin-based supramolecular peptide hydrogel. The peptide hydrogel had great effects on promoting the odontogenic differentiation of HDPCs. Self-assembling technique greatly improved the stability and biological activity of the amelogenin peptide and might exert biological functions through ERK1/2 and TGF-β/smad signaling pathways. More importantly, such hydrogel offered a suitable 3D environment for HDPCs to form mineralized nodules. Collectively, this study provided a promising strategy to promote the dentine-pulp complex regeneration.

## Funding

This work was supported by the National Science Fund for Excellent Young Scholars (T2122019), the National Natural Science Foundation of China (51973096, 51773097), the Natural Science Foundation of Tianjin City (18JCYBJC27000), the Technology Research and Development Program of Tianjin (20YFZCSY00830), the Tianjin Key Medical Discipline(Specialty) Construction Project (2021-516), the Science and Technology Project of Tianjin Health Commission (ZD20016) and the Key Laboratory of Bioactive Materials, Ministry of Education (NKBM-2019-001, NKBM-2019-002).

*Conflicts of interest statement.* The authors declare no conflict of interest. There is no financial interests or connections, direct or indirect, or other situations that might raise the question of bias in the work reported or the conclusions, implications or opinions stated – including pertinent commercial or other sources of funding for the individual author(s) or for the associated department(s) or organization(s), personal relationships, or direct academic competition.
